# Who Consults an Adult Psychiatric Emergency Department? Pertinence of Admissions and Opportunities for Telepsychiatry

**DOI:** 10.3390/medicina56060295

**Published:** 2020-06-13

**Authors:** Alessandra Costanza, Viridiana Mazzola, Michalina Radomska, Andrea Amerio, Andrea Aguglia, Paco Prada, Guido Bondolfi, François Sarasin, Julia Ambrosetti

**Affiliations:** 1 Department of Psychiatry, Faculty of Medicine, University of Geneva (UNIGE), 1211 Geneva, Switzerland; paco.prada@hcuge.ch (P.P.); guido.bondolfi@hcuge.ch (G.B.); francois.sarasin@hcuge.ch (F.S.); 2 Department of Psychiatry, ASO Santi Antonio e Biagio e Cesare Arrigo Hospital, 15121 Alessandria, Italy; 3 Department of Psychiatry, Service of Liaison Psychiatry and Crisis Intervention, Geneva University Hospitals (HUG), 1211 Geneva, Switzerland; viridiana.mazzola@hcuge.ch; 4 Faculty of Psychology, University of Geneva (UNIGE), 1206 Geneva, Switzerland; michalina.radomska@outlook.com; 5 Department of Neuroscience, Rehabilitation, Ophthalmology, Genetics, Maternal and Child Health, Section of Psychiatry, University of Genoa, 16132 Genoa, Italy; andrea.amerio@unige.it (A.A.); andrea.aguglia@unige.it (A.A.); 6 IRCCS Ospedale Policlinico San Martino, 16132 Genoa, Italy; 7 Mood Disorders Program, Tufts Medical Center, Boston, MA 02111, USA; 8 Emergency Department, Emergency Medicine Unit, Geneva University Hospitals, 1211 Geneva, Switzerland; 9 Department of Psychiatry and Emergency Department, Emergency Psychiatric Unit, Geneva University Hospitals, 1211 Geneva, Switzerland; julia.ambrosetti@hcuge.ch

**Keywords:** psychiatric emergency, suicidal behavior, depression, psychotic episode, substance use disorder, telepsychiatry, public mental health

## Abstract

Background and Objectives: Psychiatric disorders constitute frequent causes of emergency department (ED) admissions and these rates are increasing. However, referring to ED a whole range of conditions that could or should be dealt with elsewhere is imposing itself as a problematic situation. We aimed: (1) to provide a descriptive picture of the socio-demographic and diagnostic characteristics of the visits among adults at the psychiatric ED; (2) to estimate the clinical pertinence of these visits. Materials and Methods: Retrospective analysis of diagnostic/socio-demographic characteristics and clinical trajectories of patients admitted for a psychiatric condition at the adult psychiatric ED of the University Hospital of Geneva (HUG), Switzerland, during a 6-week timespan. Results: In our sample (*n* = 763 total admissions for psychiatric conditions; *n* = 702 for inclusion of patients having received a medical evaluation), depression/anxiety, suicidal behavior (SB), psychotic episode, and substance use disorder (SUD), in descending order, were the most common diagnoses for referral. Patients belonged to younger age groups (≤65 years), had a familial status other than married/in couple, and did not present an unfavorable socio-demographic profile. Concerning the pertinence for a psychiatric ED, primary diagnosis of depression/anxiety is the only variable significantly associated with different grade of degree. By the examination of the patients’ trajectory from admission to discharge, the clinical pertinence for a psychiatric ED admission existed for cases assigned to the Echelle Suisse du Tri (EST^®^) scale degree 1 (corresponding to most urgent and severe conditions), particularly for diagnoses of depression/anxiety associated with SB, SB as primary or comorbid diagnosis, and psychotic and manic/hypomanic episode. However, diagnoses of depression/anxiety without urgent and severe features (degrees 2, 3, 4) constituted the most frequent mode of presentation. Conclusions: Ambulatory and community-integrated settings could be more appropriate for the majority of patients admitted to adult psychiatric EDs. Moreover, the implementation of telepsychiatry strategies represents a very promising opportunity to offer these patients care continuity, reduce costs and filter the demand for psychiatric ED.

## 1. Introduction

In many countries, patients who access emergency departments (EDs) for psychiatric reasons are a concern for health policy leaders and emergency physicians because they present chronic and complex medical issues with high associated costs for health care delivery [1,2,3,4,5]. About one in four individuals will experience a mental illness in their lifetime [6] and the prevalence of mental illness increases as populations age. North American studies indicate that ED use for psychiatric needs has increased by 15% in the past few years and accounts for 4–12% of all ED visits [7,8,9,10]. Due to their longer length of stay, mental-health-related visits comprise a disproportionate amount of ED time. For example, in a large cohort of patients in southern California between 2009 and 2015, 8.2% of all ED visits by adults were mental-health-related, but these visits accounted for 11.2% of all ED hours utilized by adults [11]. This disproportionate consumption of ED time likely contributes to overall ED crowding which can contribute to decreased quality of care for other conditions [12,13,14,15]. Thus, the increase in psychiatric-related visits may have negative consequences for the care of all ED patients.

Psychiatric disorders requiring ED admissions are considered potentially avoidable when managed through appropriate ambulatory care [8,16,17]. The avoidance of ED visits, and repeated visits, through better continuity of care is critical for efficient ED function, especially among patients with co-morbidities [18]. Problems with access to outpatient services, and with the coordination and continuity of care after an ED visit, contribute to poor clinical outcomes and enhance the use of acute health services [19,20,21]. Research suggest that investments in primary and ambulatory health care can limit hospitalizations, with a trend for potential savings of more than four times greater than the average increase in outpatient medical costs [22]. There are emerging outpatient care paradigms that offer promise in this respect. Telepsychiatry could increase access to emergency psychiatric consultations [23,24,25]. This service modality has become increasingly common worldwide since the mid-1990s, and rapid improvements in video quality and networking capabilities have significantly improved its viability [26]. Psychiatric services via internet and mobile technologies facilitate integrated, patient-centered practices for discrete and timely care. Telepsychiatry has been evaluated on several levels and found to be effective in terms of patient acceptance and the ability to increase access to care, and with comparable outcomes to in-person services [27]. Understanding psychiatric ED user profiles and analysis of the pertinence of their admissions to EDs may contribute to developing and optimizing such alternative mental health strategies [28].

Currently the effectiveness of community- and primary-care-based interventions for limiting ED use for psychiatric illness is not well understood [29,30]. This is an area in need of focused attention, as well as carefully planned and evaluated interventions to improve patient outcomes and possibly lead to cost savings [31,32,33]. However, the amount and quality of published literature that has evaluated urgent psychiatric services is exceedingly limited, especially from European countries. 

To this end, we aimed: (1) to provide a descriptive picture of the socio-demographic and diagnostic characteristics of the visits among adults at the psychiatric ED of the University Hospital of Geneva (HUG), Switzerland; (2) to estimate the clinical pertinence of these visits. 

## 2. Materials and Methods

We retrospectively examined every admission file of patients registered at the adult division of the psychiatric ED of the HUG, Switzerland, between 1 April 2016 and 15 May 2016, by delineating a descriptive picture of their diagnostic/socio-demographic characteristics and clinical trajectory from admission to discharge. For every visit, diagnoses at admission (primary and comorbid diagnosis performed according DSM 5), demographic data (gender, age, familial and residential status, presence/absence of a social contribution, non-migrant/migrant status), patient’s access modality to ED (ambulance, police, by themselves), the type of eventual ongoing ambulatory psychiatric treatment (public multidisciplinary resources, private practice), and the decision by the psychiatrist at the discharge from ED (non-voluntary/voluntary hospitalization, returning home with or without indications for psychiatric follow-up (public multidisciplinary, private practice)) were determined. 

Data were also differentiated according to an emergency degree scale used at the HUG ED, which evaluates both the urgency and the severity of a pathological condition: the Echelle Suisse du Tri (EST^®^). The EST^®^ is one of the two screening tools recommended by the Swiss Society for Emergency Medicine and Rescue and is currently used in the three language regions of Switzerland as well as in France and Belgium. With decreasing severity, the EST^®^ scale ranges from degree 1 (a very urgent condition, dangerous to life), to 2 (a pathological situation that is not life-threatening, but which is likely to worsen quickly), to 3 (pathological situation where time is not a critical factor, the state of the patient at arrival is considered stable), to 4 (medical condition considered stable and not requiring emergency care).

Statistical analyses were based on descriptive statistical methods using SPSS version 25 and the significance was set at *p* < 0.05. The categorical variables are represented as count and percentage, while the continuous variables as mean and standard deviation. The normal distribution was assessed using Kolmogorov–Smirnov test. The Pearson’s Chi-Square was performed to evaluate the statistical significance of the frequency proportion differences across gender. Odds ratios and 95% confidence intervals were calculated using JASP Software (an open-source interface allowing to conduct classical and Bayesian statistical analyses). Subsequently, differences according to the EST^®^ were performed using Pearson’s Chi-Square and ANOVA test for categorical and continuous variables, respectively. 

This study was conducted in accordance with the Helsinki Declaration as revised in 2013 [34]. It was approved by the research ethics committee of Geneva under the registration number 2019-01533 (Approval date: 20 January 2020).

## 3. Results

A total of 763 admissions for psychiatric disorders were made at the adult psychiatric ED of the HUG during the study period. Among them, only admissions that required a psychiatric medical evaluation were included in the study (the remaining patients were evaluated by a psychiatric nurse). A final sample of 702 participants were recruited: 383 females (54.6%), 317 males (45.2%), and 2 people (0.2%) with gender disorder. The age ranged from 16 years, the minimal age to be admitted to adult psychiatric ED of the HUG, to 96 years. The mean age was 42.8 ± 18.3 (range 16–96) for females and 38.7 ± 16.5 for males (range 16–88). The differences in the socio-demographic and diagnostic characteristics are shown in Table 1 and Table 2. 

Concerning the urgency and severity estimation made at triage by the EST^®^, 96 cases (13.7%) were classified as degree 1, 312 cases (44.4%) as degree 2, 265 cases (37.8%) as degree 3, and 29 cases (4.1%) as degree 4 (Table 3). 

The 25–44 age group was the most represented (*n* = 309, 44.0%) and the ≥65 age group the least represented (*n* = 80, 11.4%). Half of the patients (*n* = 351, 50.0%) were unmarried/non-in a couple. The large majority of patients (*n* = 567, 80.8%) lived at their home, did not benefit from a social contribution (*n* = 603, 85.9%), and did not have a migrant status (*n* = 673, 95.9%) (Table 1). 

The most highly represented primary diagnosis was depression/anxiety (*n* = 310, 44.2%), followed by suicidal behavior (SB) (*n* = 82, 11.7%), psychotic episode (*n* = 77, 11.0%), substance use disorder (SUD) (*n* = 70, 10.0%), behavior disorder (*n* = 59, 8.4%), somatic problem (*n* = 47, *n* = 6.7%), behavior disorder in the elderly (*n* = 21, 3.0%), manic/hypomanic episode (13, *n* = 1.9%), psychomotor agitation (*n* = 12, 1.7%), other reasons (*n* = 8, 1.1%), and intellectual disability (*n* = 3, 0.3%). Regarding the presence of a comorbid diagnosis, SB occurred in the majority of cases (*n* = 97, 13.8%), followed by somatic problem (*n* = 53, 7.5%), behavior disorder (*n* = 41, 5.8%), SUD (*n* = 40, 5.7%), psychomotor agitation (*n* = 29, 4.1%), depression/anxiety (*n* = 14, 2.0%), behavior disorder in the elderly (*n* = 5, 0.7%), psychotic and manic/hypomanic episode (*n* = 2 for both, 0.3%), and other reasons (*n* = 2, 0.3%). No cases of comorbid diagnosis of intellectual disability was registered (Table 1).

Regarding to the differences of the diagnostic characteristics across gender, females had more depression/anxiety (62.9% vs. 37.1%) and behavior disorder in the elderly (76.2% vs. 23.8%) while males had significantly more SB (57.3% vs. 42.7%) and behavior disorder (62.7% vs. 37.3). The other not significant differences in terms of the first diagnostic characteristics across gender are displayed in Table 2.

According to the EST^®^, primary diagnosis and no other features (socio-demographic or social) is the only variable significantly associated with a different grade of degree (Table 3). With regard to post-hospital trajectories and outcomes differentiated by severity of mental illness, for degree 1, after consultation at the psychiatric ED, 36 patients (37.5%) were hospitalized. Regarding non-voluntary hospitalizations (*n* = 25, 69%), the diagnoses of psychotic episode (*n* = 13, 52%) and maniac/hypomanic episode (*n* = 5, 20%, constituting the totality of the cases with this diagnosis) were the most represented. Psychomotor agitation accounted for one non-voluntary hospitalization. The large majority of patients with SB as principal or accessory diagnosis were hospitalized (*n* = 30, 81%). For degree 2, 13 (61.9%) women were followed-up by a psychiatrist (*n* = 8, 61.5% in psychiatric private practice), 25 (83.3%) returned home, of whom ten (33.3%) were sent to a private psychiatrist. Eight men (32%) came by themselves while undergoing outpatient psychiatric treatment (*n* = 3, 37.5% in private practice) and eight came by themselves and did not have ongoing outpatient psychiatric treatment. Twenty-two men (88%) returned home, including 14 (56%) with the indication to be monitored in multidisciplinary public structures, and five (20%) were referred to a private psychiatrist. For degree 3, regarding women: 33 (55%) came by themselves and did not a have ongoing psychiatric treatment; and 49 (81.7%) returned home, of whom 22 (44.9%) were referred to private psychiatrists. With regard to men: 20 (58.8%) had no ongoing psychiatric treatment, seven (20.6%) were followed-up in multidisciplinary public structures, and 28 (82.4%) returned home after their ED assessment. For degree 4, 22 cases (75%) did not have an ongoing psychiatric treatment, and 24 cases (82%) returned home (Table 3). Further details on diagnostic /socio-demographic characteristics with the four-triage degrees are presented in the Supplementary Online Material.

## 4. Discussion

In our sample, the diagnoses of depression/anxiety, SB, psychotic episode, and SUD (in descending order) are the most common reasons for referral to the adult psychiatric ED. SB and psychotic episodes accounted for approximately the same proportion of patients. SB was also the most common accessory diagnosis. The distribution of these diagnoses is consistent with the general prevalence of mental disorder within the Swiss population [35] and, except for SB, with the literature [8,36,37]. The finding related to SB is also in agreement with its general prevalence in Switzerland, which is higher than most European countries [38,39,40]. From a socio-demographic point of view, no significant differences in gender were observed, as previously described [36], but some recent works have reported an excess of male patients [28]. Females had more depression/anxiety and behavior disorder among the elderly, while males had significantly more SB and behavior disorder. The patients tended to belong to younger age groups (≤65 years), in line with the literature [28,36]. They were mostly single, separated/divorced, or widowed, conditions that may reflect a lack of personal and social support [36]. In contrast to other reports [8,28,36,41], our study group did not have an overall unfavorable social profile (they tended to live at their home, did not benefit from a social contribution, and were not migrants). However, the generalizability of this finding might depend on country-specific health insurance systems. For example, in Switzerland, contrary to the U.S., a mandatory insurance system covers psychiatric ED care to the same level as treatment in other specialized psychiatric settings, and patients lacking financial resources are generally free to choose a treatment option other than EDs [42]. Moreover, in Switzerland most of the patients have direct access to psychiatrists as the first point of contact for mental health issues, compared to the “gatekeeper system” which is the predominant form of mental health care in the U.S. [43].

First diagnosis of depression/anxiety is the only variable significantly associated with different grade of degree. By the examination of the patients’ trajectory from admission to discharge, clinical pertinence for a psychiatric ED admission existed for cases assigned to the most severe EST^®^ scale degree (degree 1), particularly for diagnoses of depression/anxiety associated with SB, SB as first or accessory diagnosis, and psychotic and manic/hypomanic episodes. However, diagnoses of depression/anxiety without urgent and severe features (degrees 2, 3, 4) constituted the most frequent mode of presentation. 

In detail, among the consultations classified as degree 1 (corresponding to most urgent and severe conditions), the primary diagnosis of depression/anxiety was found in the majority of the cases and associated with SB in one third of the cases. Considering both primary and comorbid diagnoses, SB accounted for the majority of the cases. The psychomotor agitation diagnosis, classified as degree 1 by definition, was surprisingly underrepresented and a small number of psychomotor agitation diagnoses emerged among the non-voluntary hospitalizations. One possible explanation may be that at least some of the agitations were designated as a psychotic and manic/hypomanic episode. With regard to clinical trajectory, a large majority of patients: had a diagnosis of depression/anxiety associated with SB; had SB as a first or accessory diagnosis; had a psychotic and manic/hypomanic episode; arrived by ambulance or police; and were hospitalized after the ED assessment. This may lead to the inference that clinical pertinence with an ED visit exists for degree 1, in particular for these respective diagnoses.

Degrees 2 and 3 (corresponding to less urgent and severe conditions than degree 1) accounted for the large majority of the visits. As with degree 1, the most highly represented primary diagnosis was depression/anxiety. However, the proportion of patients with this diagnosis that came by themselves to the ED and having an ongoing outpatient psychiatric treatment was higher compared to degree 1. At the post-ED orientation, the large majority of degree 2 and 3 patients were not hospitalized. Surprisingly, the association of depression/anxiety with a comorbid diagnosis of SB accounted for more cases among degree 2 visits than degree 1, but its clinical severity was lower. Some differences between the patients’ gender with depression/anxiety emerged. Most of these patients were women, had an ongoing outpatient psychiatric treatment, and, after ED assessment, returned home and were referred to a private psychiatrist. Furthermore, they tended to be married/in couple and did not have an unfavorable socio-demographic profile. Men were less likely to have ongoing outpatient psychiatric treatment and were more often referred to public ambulatory multidisciplinary structures offering the possibility of overnight stays, which would suggest clinical diagnoses that were perceived as more severe and/or complicated. In contrast to women, they more frequently had a familial status other than married/in couple and presented a slightly more unfavorable socio-demographic profile. Patients with the described trajectory configure an excessive high percentage for an ED.

Degree 4 consultations accounted for a minority of cases, which tended to have mild depression/anxiety diagnoses. Their clinical trajectories were not appropriate for an ED. 

In summary, visits receiving the diagnosis of depression/anxiety without urgent and severe features (degrees 2, 3, and 4) were the most frequent mode of presentation to our adult psychiatric ED. An ambulatory and community-integrated setting could be more appropriate for patients belonging to this group. In particular, they could benefit from access to ambulatory facilities that can provide for a rapid inclusion of unscheduled cases [8,16,17,28,44]. 

Another interesting opportunity for patients who inadequately consult psychiatric ED, is the use of telepsychiatry. The latter, also known as telemental health, is commonly defined as “the delivery of mental health care in the form of live and interactive videoconferencing” [45]. The psychiatrist can be summoned promptly through videoconferencing to evaluate the patient and then recommend treatment options. After two decades of debate around the rationale, use, andimpact of telepsychiatry [46], the World Psychiatric Association (WPA)-Lancet Psychiatry Commission on the Future of Psychiatry has advocated the implementation of digital psychiatry in the present and the very near future [47]. Using the right recommendations and protocols [45], on-demand telepsychiatry has been proven to offer a closer continuity of care and reduce ED pressure, discharge times, psychiatric hospitalization rates, and it is associated with good outcomes and high patient satisfaction [48]. In particular, the majority of mild psychiatric conditions can be managed rapidly, in contrast with the finding that in EDs psychiatric patients wait on average three times longer compared to other patients for a medical evaluation [49]. Telepsychiatry also represents a cost-effective intervention [50]. Due to the increasing use of technology (i.e. smartphones), the use of technology-based interventions may be the most adequate method of care for psychiatric patients presenting mild clinical presentations. Beyond the reluctance that psychiatrists can understandably manifest towards technological devices for fear that they will transform the type of interpersonal relationship or even accentuate the stigma, telepsychiatry can instead be considered a means to remotely support patients to cope closer with their loneliness, feelings of diminished social connectedness, hopelessness, and helplessness [51,52,53]. 

## 5. Limitations

This study had several limitations. First, it was a naturalistic observational study and, consequently, an experimental control was not included, thus precluding inferential conclusions from being drawn. Second, only a short period of observation (six weeks) was monitored, and this limited time span may introduce a potential bias concerning the type of diagnosis that can be more frequent during this specific period. Third, by using the admission files diagnoses, we did not distinguish various types of personality disorders (in particular, the diagnosis of borderline disorder personality). On the other hand, the strengths of this study were: having a snapshot that included all adult psychiatric ED admissions during the study period (day and night, weekdays and holidays); its location (a large metropolitan area representing the entire Geneva canton); and the concerned population (patients attending a public hospital). These latter factors may facilitate the generalizability of the findings to other public psychiatric settings, as well their comparison. 

## 6. Conclusions

The obtained data may inform possible alternative strategies to deal with a whole range of psychiatric patients that can be treated elsewhere than in EDs. Ambulatory and community-integrated settings could be more appropriate for the majority of these patients. Moreover, the implementation of telepsychiatry strategies also represents a very promising opportunity to offer these patients a closer care continuity, reduce costs, and filter the demand for psychiatric ED. The involvement of all key clinicians, along with organizational and technological support, is necessary to guarantee more success to telepsychiatry in ED. 

## Figures and Tables

**Table 1:** Characteristics of the patients included in the study (*n* = 702).

***Socio-Demographic Characteristics***	***n***	***%***
**Gender**		
Females	383	54.6
Males	317	45.2
Gender Disorder	2	0.2
**Age** (years)		
16–24	137	19.5
25–44	309	44.0
45–64	175	24.9
≥65	80	11.4
**Familial status**		
Married/in couple	209	29.8
Unmarried/non-in couple	351	50.0
Separated/divorced	108	15.4
Widowed	32	4.6
**Residential status**		
Private residence	567	80.8
Nursing home, foster home, hotel	72	10.3
Homeless	31	4.4
**No social contribution**	603	85.9
**No migrant status**	673	95.9
***Diagnostic Characteristics***		
**Primary diagnosis**		
Depression/anxiety	310	44.2
Suicidal Behavior	82	11.7
Psychotic episode	77	11.0
Substance Use Disorder	70	10.0
Behavior disorder	59	8.4
Somatic problem	47	6.7
Behavior disorder in the elderly	21	3.0
Manic/hypomanic episode	13	1.9
Psychomotor agitation	12	1.7
Intellectual disability	3	0.3
Other reason	8	1.1
**Comorbid diagnosis**		
Suicidal behavior	97	13.8
Somatic problem	53	7.5
Behavior disorder	41	5.8
Substance use disorder	40	5.7
Psychomotor agitation	29	4.1
Depression/anxiety	14	2.0
Behavior disorder in the elderly	5	0.7
Psychotic episode	2	0.3
Manic/hypomanic episode	2	0.3
Other reason	2	0.3

Missing data: age *n* = 1; familial status *n* = 2; residential status *n* = 32; social contribution *n* = 2.

**Table 2:** Primary diagnosis across gender.

*Primary Diagnosis*	*n* (%)	χ^2^	*p*	OR	CI 95% (Lower; Upper)
Depression/anxiety		15.06	<0.001	−0.60	−0.90; −0.03
Females	195 (62.9)				
Males	115 (37.1)				
Suicidal behavior		5.01	<0.01	−0.61	0.05; 0.97
Females	35 (42.7)				
Males	47 (57.3)				
Psychotic episode		2.12	n.s.	0.31	−0.11; 0.81
Females	34 (44.1)				
Males	43 (55.9)				
Substance use disorder		0.35	n.s.	0.16	−0.22; 0.61
Females	36 (51.4)				
Males	34 (48.6)				
Behavior disorder		7.90	<0.01	0.77	0.22; 1.32
Females	22 (37.3)				
Males	37 (62.7)				
Somatic problem		0.27	n.s.	0.16	−0.43; 0.75
Females	24 (51.1)				
Males	23 (48.9)				
Behavior disorder in the elderly		4.03	<0.05	−1.01	−2.02; 0.01
Females	16 (76.2)				
Males	5 (23.8)				
Manic/hypomanic episode		1.13	n.s.	−0.63	−1.82; 0.55
Females	9 (69.2)				
Males	4 (30.8)				
Psychomotor agitation		0.84	n.s.	0.53	−0.62; 1.69
Females	5 (41.7)				
Males	7 (58.3)				

OR = Odds Ratio n.s. = non-significant value.

**Table 3:** Socio-demographic characteristics and first diagnoses differentiated according to the Echelle Suisse du Tri (EST®).

	Degree 1 (*n* = 96)	Degree 2 (*n* = 312)	Degree 3 (*n* = 265)	Degree 4 (*n* = 29)	*p*
**Gender**, *n* (%)					
Female	50 (52.1)	166 (53.2)	153 (57.7)	14 (48.3)	n.s.
Male	46 (47.9)	145 (46.5)	111 (41.9)	15 (51.7)	
Patient with gender disorder	0 (0.0)	1 (0.3)	1 (0.4)	0 (0.0)	
**Age** (years), mean ± SD	39.9 ± 16.4	41.2 ± 18.1	40.9 ± 17.9	40.8 ± 15.3	n.s.
**Familial status**, *n* (%)					
Married/in couple	26 (27.1)	79 (25.3)	92 (34.7)	12 (41.4)	n.s.
Unmarried/non-in couple	49 (51.0)	173 (55.4)	114 (43.0)	15 (51.8)	
Separated/divorced	18 (18.8)	47 (15.1)	42 (15.8)	1 (3.4)	
Widowed	2 (2.1)	13 (4.2)	16 (6.0)	1 (3.4)	
**Residential status**, *n* (%)					
Private residence	79 (82.3)	259 (83.0)	210 (79.2)	19 (65.5)	n.s.
Nursing home, foster home, hotel	8 (8.3)	30 (9.6)	29 (10.9)	5 (17.2)	
Homeless	4 (4.2)	8 (2.6)	15 (5.7)	4 (13.8)	
**Social contribution**, Yes, *n* (%)	10 (10.4)	46 (14.7)	37 (14.0)	4 (14.0)	n.s.
**Migrant status**, Yes, *n* (%)	5 (5.2)	10 (3.2)	14 (5.3)	0 (0.0)	n.s.
**Primary diagnosis**, *n* (%)					<0.001
Depression/anxiety	34 (35.4)	114 (36.6)	148 (55.9)	14 (48.3)	
Suicidal behavior	15 (15.6)	49 (15.7)	16 (6.0)	2 (6.9)	
Psychotic episode	15 (15.6)	45 (14.4)	16 (6.0)	1 (3.4)	
Substance use disorder	8 (8.3)	39 (12.5)	21 (7.9)	2 (6.9)	
Manic/hypomanic episode	4 (4.2)	6 (1.9)	3 (1.1)	0 (0.0)	
Psychomotor agitation	6 (6.3)	6 (1.9)	0 (0.0)	0 (0.0)	
Behavior disorder	10 (10.4)	21 (6.7)	24 (9.1)	4 (13.8)	
Behavior disorder in the elderly	1 (1.0)	13 (4.2)	6 (2.3)	1 (3.4)	
Intellectual disability	0 (0.0)	1 (0.3)	1 (0.4)	1 (3.4)	
Somatic problem	3 (3.1)	15 (4.8)	25 (9.4)	4 (13.8)	
Other reasons	0 (0.0)	3 (1.0)	5 (1.9)	0 (0.0)	

Missing data: age *n* = 1; familial status *n* = 2 (degree 2—*n* = 1, degree 3—*n* = 1); residential status *n* = 32 (degree 1—*n* = 5, degree 2—*n* = 15, degree 3—*n* = 11, degree 4—*n* = 1); social contribution *n* = 2 (degree 2—*n* = 1, degree 3—*n* = 1). n.s. = non-significant value.

## Supplementary Materials

The following are available online at https://www.mdpi.com/1010-660X/56/6/295/s1. Details on diagnoses, socio-demographic characteristics, and patients’ trajectory data differentiated in accordance with the Echelle Suisse du Tri (EST^®^). Degree 1: 96 cases (13.7 %) were classed as degree 1. There were no relevant differences between females (*n* = 50, 52%) and males (*n* = 46, 48%). All age classes were represented (mean age: 39.9 ± 16.4, range: 16-86), but a large majority of the patients (80%) were aged ≤ 50 years. Depression/anxiety was diagnosed the most often (*n* = 34, 35.4%). The other most represented diagnoses were, in descending order: suicidal behavior (SB) and psychotic episode (*n* = 15 for each, 15.6%); behavior disorder (*n* = 10, 10.4%); substance use disorder (SUD) (*n* = 8, 8.3%); and psychomotor agitation (*n* = 6, 6.3%). Fifty-three (56%) patients had an accessory diagnosis, represented by SB in the majority of cases (*n* = 22, 29%). Among them, 17 (77%) were women and five (22.7) were men and were referred by private psychiatrists in almost half of the cases (*n* = 8, 42%). Forty-four patients (45.8%) were admitted to psychiatric ED by ambulance, and 15 (15.6 %) by police. Degree 2: 312 cases (44.4%) were classified as degree 2, without relevant differences between females (*n* = 165, 53.2%) and males (*n* = 146, 46.8%); one patient had gender disorder. All age classes were represented (mean age: 41.2 ± 18, range: 16–90 years), but the large majority of patients (80%) were aged ≤ 55 years. Most of the cases (*n* = 114, 36.5%) had the diagnosis of depression/anxiety. The other most represented diagnoses were, in descending order: SB (*n* = 49, 15.7 %); psychotic episode (*n* = 45, 14.4 %); SUD (*n* = 39, 12.5 %); and behavior disorder (*n* = 21, 6.7%). The 114 cases of depression/anxiety diagnoses were examined in greater detail. Among them, 46 cases (40.4%) were associated with SB, and 28 (60.9%) cases were women. The other two most represented comorbid diagnoses were somatic (seven cases) and SUD (three cases). Fifty-five cases of depression/anxiety (48.2%) had no comorbid diagnosis. In this category, 30 patients were women (54.5%) and 25 were men (45.5%). Twenty-one women (70%) came by themselves. From a socio-demographic point of view, 12 women (40%) were not married/non-in couple, 11 (36.7%) were married/in couple, 6 (20%) were divorced. Twenty-four (80%) did not have a social contribution, 28 (93.3%) lived in their home, 1 patient lived in a foster home, and 1 was homeless; none were migrants. With regard to men: 22 (88%) were not married/non-in couple and 3 (12%) were married/in couple. Twenty-three (92%) did not have a social contribution, 19 (76%) lived in their home, 5 (20%) lived in foster homes, and 2 (8%) were migrant. Altogether, patients with depression/anxiety as the sole diagnosis returned home after their assessment in the ED in the large majority of the cases (*n* = 47, 85%). Degree 3: 265 cases (37.8 %) were classified as degree 3. One-hundred and fifty-three cases (57%) were females, and 1 patient had a gender disorder. All age classes were represented (mean age: 41 ± 17.72, range: 16–90 years), but the large majority of patients (80%) were aged ≤ 55 years. Most of the cases (*n* = 148; 55.8%) had the diagnosis of depression/anxiety. The other most represented diagnoses were, in descending order: somatic problem (*n* = 25; 9.4%); behavior disorder (*n* = 24, 9.1%); SUD (*n* = 21; 7.9%); and SB and psychotic episode (*n* = 16 for each, 6%). The 148 cases of depression/anxiety were examined in greater detail. Among them, 28 cases (18.9%) were associated with SB, and 18 cases (64.3%) were women. The other two most represented comorbid diagnoses were somatic (10 cases) and SUD (8 cases). 94 cases of depression/anxiety (62.9%) had no comorbid diagnosis. In this category, 60 (64%) were women and 34 (36%) were men. From a socio-demographic point of view: 36 women (60%) were married/in couple and did not have a social contribution, 3 lived in foster homes and hotels, and one was a migrant. Fourteen men (41.2%) were married/in couple (the remaining: not married/non-in couple, divorced/separated, widowed), 5 had a social contribution, 3 lived in hotels, 3 were homeless, and 5 were migrant. Altogether, patients with depression/anxiety as the sole diagnosis returned home after their assessment in the ED in the large majority of cases (*n* = 77, 81.91%); degree 4: 29 cases (4.13%) were included, half of which were females (*n* = 14) and half were males (*n* = 15); all ages were represented, but 80% of cases were ≤ 51 years (mean age: 40.8 ± 15.3). Depression/anxiety was the first diagnosis in 14 cases (48%). Among them, there was no associated SB, and for the 4 patients who did have a comorbid diagnosis, these were classified as a somatic problem. For the 10 patients with depression/anxiety as the only diagnosis, half were women (*n* = 5) and half were men (*n* = 5).
