# Supplementary material for: Who Consults an Adult Psychiatric Emergency Department? Pertinence of Admissions and Opportunities for Telepsychiatry
Source: Medicina (Kaunas). 2020 Jun 13;56(6):295. doi: 10.3390/medicina56060295 (PMC7353920; doi:10.3390/medicina56060295)
Supplement: Supplementary file 1 [file medicina-56-00295-s001.pdf]

Word count: 984

Supplementary Online Materials

Details on diagnoses, socio-demographic characteristics, and patients' trajectory data differentiated in accordance with the triage HUG urgency and severity scale.

Degree 1. 96 cases (13.67 %) were classed as degree 1. There were no relevant differences between females (n=50, 52%) and males (n=46, 48%). All age classes were represented (mean age: 39.9 ± 16.4, range: 16-86), but a large majority of the patients (80%) were aged ≤ 50 years. Depression/anxiety was diagnosed the most (n=34, 35.4%). The other most represented diagnoses were, in descending order: SB and psychotic episode (n=15 for each, 15.6%); behavior disorder (n=10, 10.4%); SUD (n=8, 8.3%); and psychomotor agitation (n=6, 6.3%). 53 (56%) patients had an accessory diagnosis, represented by SB in the majority of the cases (n=22, 29%). Among them, 17 (77%) were women and 5 (22.7) were men, and referred by private psychiatrists in almost half of the cases (n= 8, 42%). 44 patients (45.8%) were admitted to psychiatric ED by ambulance, and 15 (15.6 %) by police. After consultation at the psychiatric ED, 36 patients (37.5%) were hospitalized. Regarding non-voluntary hospitalizations (n=25, 69%), the diagnoses of psychotic episode (n=13, 52%) and maniac/hypomanic episode (n=5, 20%, constituting the totality of the cases with this diagnosis) were the most represented; psychomotor agitation accounted for 1 non-voluntary hospitalization. The large majority of patients with SB as principal or accessory diagnosis were hospitalized (n=30, 81%).

Degree 2. 312 cases (44.4%) were classified as degree 2, without relevant differences between females (n=165, 53.2%) and males (n=146, 46.8%); one patient had gender disorder. All age classes were represented (mean age: 41.2 ± 18, range: 16-90 years), but the large majority of patients (80%) were aged ≤ 55 years. Most of the cases (n=114, 36.5%) had the diagnosis of depression/anxiety. The other most represented diagnoses were, in descending order: SB (n=49, 15.7 %); psychotic episode (n=45, 14.4 %); SUD (n=39, 12.5 %); and behavior disorder (n=21, 6.7%). The 114 cases of depression/anxiety diagnoses were examined in greater detail. Among them, 46 cases (40.4%) were associated with SB, and 28 (60.9%) cases were women. The other two most represented accessory diagnoses were somatic (7 cases) and SUD (3

cases). 55 cases of depression/anxiety (48.2%) had no accessory diagnosis. In this category, 30 patients were women (54.5%) and 25 were men (45.5%). 21 women (70%) came by themselves, including 13 (61.9%) who were followed-up by a psychiatrist (n=8, 61.5% in private practice). 25 among them (83.3%) returned home, of whom 10 (33.3%) were sent to a private psychiatrist. 8 men (32%) came by themselves while undergoing outpatient psychiatric treatment (n=3, 37.5% in private practice) and 8 came by themselves and did not have ongoing outpatient psychiatric treatment. 22 men (88%) returned home, including 14 (56%) with the indication to be monitored in multidisciplinary public structures, and 5 (20%) were referred to a private psychiatrist. From a socio-demographic point of view, 12 women (40%) were not married/non-in couple, 11 (36.7%) were married/in couple, 6 (20%) were divorced. 24 (80%) did not have a social contribution, 28 (93.3%) lived in their home, 1 patient lived in a foster home, and 1 was homeless; none were migrant. With regard to men: 22 (88%) were not married/non-in couple and 3 (12%) were married/in couple. 23 (92%) did not have a social contribution, 19 (76%) lived in their home, 5 (20%) lived in foster homes, and 2 (8%) were migrant. Altogether, patients with depression/anxiety as the sole diagnosis returned home after their assessment in the ED in the large majority of the cases (n=47, 85%).

Degree 3. 265 cases (37.8 %) were classified as degree 3. 153 cases (57%) were females, and 1 patient had gender disorder. All age classes were represented (mean age: 41 ± 17.72, range: 16-90 years), but the large majority of patients (80%) were aged ≤ 55 years. Most of the cases (n=148; 55.8%) had the diagnosis of depression/anxiety. The other most represented diagnoses were, in descending order: somatic problem (n=25; 9.4%); behavior disorder (n=24, 9.1%); SUD (n=21; 7.9%); and SB and psychotic episode (n=16 for each, 6%). The 148 cases of depression/anxiety were examined in greater detail. Among them, 28 cases (18.9%) were associated with SB, and 18 cases (64.3%) were women. The other two most represented accessory diagnoses were somatic (10 cases) and SUD (8 cases). 94 cases of depression/anxiety (62.9%) had no accessory diagnosis. In this category, 60 (64%) were women and 34 (36%) were men. Regarding women: 33 (55%) came by themselves and did not a have ongoing psychiatric treatment; and 49 (81.7%) returned home, of whom 22 (44.9%) were referred to private psychiatrists. With regard to men: 20 (58.8%) had no ongoing psychiatric treatment, 7 (20.6%) were followed-up in multidisciplinary public structures, and 28 (82.4%) returned home after their ED assessment. From a socio-demographic point of view: 36 women (60%) were married/in couple and did not have a social contribution, 3 lived in foster homes and hotels, and

one was a migrant. 14 men (41.2%) were married/in couple (the remaining: not married/non-in couple, divorced/separated, widowed), 5 had a social contribution, 3 lived in hotels, 3 were homeless, and 5 were migrant. Altogether, patients with depression/anxiety as the sole diagnosis returned home after their assessment in the ED in the large majority of cases (n=77, 81.91%).

Degree 4. 29 cases (4.13%) were included, half of which were females (n=14) and half were males (n=15); all ages were represented, but 80% of cases were ≤ 51 years (mean age: 40.8 ± 15.3). 22 cases (75%) did not have an ongoing psychiatric treatment, and 24 cases (82%) returned home. Depression/anxiety was the first diagnosis in 14 cases (48%). Among them, there was no associated SB, and for the 4 patients who did have an accessory diagnosis, these were classified as a somatic problem. For the 10 patients with depression/anxiety as the only diagnosis, half were women (n=5) and half were men (n=5).

Supplementary Materials: The following are available online at www.mdpi.com/xxx/s1.
